# Trauma‐Informed Individual Placement and Support in a Youth Community Service in Regional Australia: Trauma‐Exposed Young People's Experiences

**DOI:** 10.1111/eip.70271

**Published:** 2026-10-16

**Authors:** Wilma Peters, Ilias Kamitsis, Fatima Ibrahim, Jannette Kavanagh, Gina Chinnery, Sarah Bendall

**Affiliations:** ^1^ Orygen Parkville Victoria Australia; ^2^ Centre for Youth Mental Health The University of Melbourne Melbourne Victoria Australia

**Keywords:** employment support, individual placement and support, trauma, trauma‐informed care, youth mental health

## Abstract

**Introduction:**

Individual Placement and Support (IPS) is an evidence‐based intervention to support people with severe mental ill health to engage in work or education. However, how it should be integrated into a service for young people with high levels of exposure to trauma is unclear. This project involved the development of a trauma‐informed training and supervision model to augment IPS training and the implementation of the trauma‐informed‐IPS (TI‐IPS) training and supervision model TI‐IPS into a youth community service in regional Australia. The aim of the research project was to understand how young people experienced receiving the TI‐IPS model.

**Methods:**

TI‐IPS was implemented at Veritas House. Ten trauma‐exposed young people who had received TI‐IPS participated in in‐depth qualitative interviews about their experiences. Results were analysed using Reflexive Thematic Analysis.

**Results:**

Young people described how important trust in the TI‐IPS worker was and how trust supported them in being motivated to look for work. They also described feeling safe interacting with their IPS worker. Young people talked about trauma with their IPS workers in a brief and contained way and experienced this as helpful and supportive.

**Conclusions:**

The trauma‐informed and IPS models of care can be integrated successfully in youth services. Young people highly value the relationship with their worker when undertaking TI‐IPS.

## 1 Introduction

Exposure to trauma is common among young people, with studies estimating 62%–65% will be exposed to at least one traumatic event before age 18 (Mathews et al. 2023; Swedo et al. 2024). Exposure to traumatic events is more common among specific groups such as young people in out‐of‐home care, those who experience homelessness, refugees, people experiencing family and domestic violence, Aboriginal and Torres Strait Peoples, LGBTQI+ people, and those involved in the justice system (Bendall, Phelps, et al. 2018). A large body of research shows that, alongside poorer mental‐health outcomes, trauma‐affected children (or young people) have poor outcomes on a wide range of educational and economic indicators (Misiak et al. 2022). Childhood trauma appears to negatively impact early schooling, increasing absenteeism, drop‐out, grade repetition, and lower academic achievement (Metzler et al. 2017). Trauma‐exposed young people are also less likely to complete secondary schooling and attend or graduate from tertiary institutions compared to their non‐trauma‐exposed counterparts (Stewart‐Tufescu et al. 2022). In early adulthood, trauma‐exposed individuals are at an increased risk of experiencing employment instability and unemployment (Bunting et al. 2018), homelessness (Barnes et al. 2021; Davies and Allen 2017), and being or having been on employment disability payments (Doidge et al. 2017; Sansone et al. 2005).

Like their peers, trauma‐exposed young people want to work and/or study (Adamus et al. 2024; Metzler et al. 2017). However, despite high rates of trauma exposure reported by those accessing youth mental health services, vocational programs integrated within mental health services have yet not attended to the impacts of trauma within their service models (Cross 2020). Addressing trauma has been identified as a critical gap across youth mental health service delivery (Bendall, Phelps, et al. 2018). This has led to service users reporting a lack of understanding of their experiences and re‐traumatisation from lack of responsive care (Ellinghaus et al. 2021; Truss et al. 2022). It can also lead to vicarious trauma and burn‐out in service providers, who describe not understanding or knowing how to talk about trauma with the young people they work with, and thus avoiding it (Gairns et al. 2015). Trauma‐informed employment and vocational support programs thus not only stand to benefit young people directly but also their support workers. It also holds significant potential to reduce the incidence of childhood trauma by addressing economic disadvantage early (Corbett et al. 2024; Skiba 2020).

Trauma‐informed care (TIC) and Individual Placement and Support (IPS) are two service provision models that may be able to be combined to better meet the needs of trauma‐affected young people with vocational and educational difficulties. IPS is an evidence‐based vocational rehabilitation intervention developed to support people with severe mental ill‐health in achieving educational and competitive employment outcomes (Drake 1998). IPS supplanted traditional pre‐employment skills and training programs with an approach that placed client preference and rapid placement in competitive employment at the core of its activities. Numerous randomised controlled trials of IPS demonstrate that it is an effective intervention (de Winter et al. 2022). IPS is effective in supporting young people with poor mental health, including those experiencing psychosis (Bond et al. 2023; Drake 1998; Killackey et al. 2013, 2019; Nicholas et al. 2024). IPS is a strength‐based model underpinned by eight core principles of: focus on competitive employment, eligibility based on client choice, integration of rehabilitation and mental health services, attention to participant preferences, personalised benefits counselling, rapid job search, systematic job development, time‐unlimited and individualised support (Bond et al. 2012).

TIC has emerged as a holistic care model well‐suited to meeting the needs of trauma‐exposed individuals across service settings (Berring et al. 2024; Saunders et al. 2023). Unlike trauma‐focused therapies, which seek to directly ameliorate the symptoms of post‐traumatic stress disorder, trauma‐informed care is conceptualised as a broader approach to providing service environments that address the specific needs and sensitivities of individuals who have experienced trauma. A widely used definition of trauma‐informed care is the Substance Abuse and Mental Health Services Administration's (SAMHSA) ‘Four R's’ of *Realising* the widespread impact of trauma, *Recognising* the signs and symptoms of trauma among clients and staff, *Responding* by integrating knowledge about trauma into practice and policy, and proactively resisting *Retraumatisation* (SAMHSA 2014). TIC operates according to 6 key principles: safety, trustworthiness and transparency; peer support; collaboration and mutuality; empowerment voice and choice; attention to race, gender, and historical issues (SAMHSA 2014). These descriptors operate at a high level that can be applicable to many service systems. TIC in youth mental health services has been shown to operate at both the system level (to include, for example, interagency collaboration, service provider training, safety) and at the trauma‐specific practice level (which includes screening, psycho‐education therapeutic interventions; Bendall et al. 2021). There is a need to integrate TIC into specific practice elements of the youth mental health model (such as IPS) to ensure that TIC is operating in all parts of a trauma‐informed youth service. This integration seldom occurs as TIC training is often delivered at the systems level with the assumption that this will filter down to practice. However, it is known from implementation science research that training alone rarely leads to practice change (Rapport et al. 2022).

TIC and IPS have many features in common. Both models have gained wide reach, and as a consequence, have been adapted in differing settings (Adams et al. 2023; Bendall et al. 2021; Ellison et al. 2015; Ferguson et al. 2012). To our knowledge, these two models have not been integrated into youth mental health or residential care in Australia or the world. As such, and building on the research previously undertaken to understand TIC in youth mental health settings (Bendall et al. 2021) and on Trauma Informed Psychotherapy for Psychosis (Bendall, Alverez‐Jimeneza, et al. 2018), Orygen has developed a model for Trauma‐informed IPS (TI‐IPS) training and implementation. The current research project is part of an Australian Government Local Jobs Program funded a 3‐year implementation project (2021–2025) to embed IPS workers in Veritas House, a community‐based support service for young people in out‐of‐home care in regional New South Wales (NSW), Australia. As the young people accessing this service had extremely high exposure to trauma, and the service currently operated from a TIC perspective, our team developed and delivered a TIC training and support package that was adjunctive but interconnected with standard IPS training and support. This was undertaken to ensure that the new IPS service was consistent with the organisation's trauma‐informed focus. The aim of the research study was to explore, through in‐depth interviews with young people receiving Veritas services, the experience of taking part in TI‐IPS. This allowed us to gain an understanding of what young trauma‐exposed service users found important and helpful within TI‐IPS to begin to elucidate a theoretical model of a TI‐IPS for further research and improvement to the training and support package.

## 2 Methods

### 2.1 Research Context

TI‐IPS was implemented at Veritas House, NSW. Veritas House is a community organisation providing a range of flexible accommodation and support services, including out‐of‐home care to children, young people, and foster carers in rural NSW. Trauma exposure is common among children and young people accessing services such as those provided at Veritas House. As such, in accordance with National directives (Health 2023), Veritas identified a need to implement trauma‐informed vocational and educational services given the success of IPS implementation within youth mental health services.

### 2.2 Training Development and Implementation

The training was developed by W.P. and S.B., both experts in TIC training and implementation in youth mental health. W.P. developed knowledge in IPS by attending trainings and reviewing literature on IPS and IPS in youth mental health settings. The literature was reviewed on trauma‐informed training programs and employment/educational service provision in out‐of‐home care/youth homelessness. In collaboration with two young people from the youth advisory group of an Orygen‐based youth mental health service and the IPS expert in our research team (CG), W.P. completed evidence mapping to understand the philosophy and principles of TIC and IPS and their overlaps (e.g., strength‐based approach). TIC in youth mental health requires actions at three levels of service delivery—the system (which includes service culture and policy), screening and assessment of trauma, and trauma‐informed practice elements (Bendall, Phelps, et al. 2018; Bendall, Alverez‐Jimeneza, et al. 2018; Bendall et al. 2021).

As Veritas already had measures to embed trauma‐informed care at the service level, and trauma assessment was not within IPS workers' remit, the training focused on IPS practice. The training included four broad elements. The first was understanding trauma using the three E's: event(s), experience and effect (SAMHSA 2025) and introduction to a suite of online youth‐friendly resources to extend IPS workers' understanding of trauma and that IPS workers could give to, or work through with, the young people with whom they worked. The second was introduction to the key principles of TIC and how they applied across the phases of IPS (engaging and referring clients, vocational profiling, proactive employer engagement, job matching and securing employment, and in‐work support). The third element was training on how to manage talking about trauma if young people brought it up within IPS. This involved using the *Look, Listen, Link* model of psychological first aid (Wang et al. 2021). IPS workers were trained on how to notice the young person's emotions in session (*Look*), to *listen* to a young person if they brought up their trauma, and then to refer them to new or existing clinical supports to deal directly with trauma if needed (*Link*). This gave these non‐clinically trained workers a framework to attend to trauma without having to go beyond the expectations of non‐clinical staff. The fourth part of the training was in how to manage vicarious trauma. The training has a practical focus through the collaborative exploration of a case study and trainees generating and recording specific trauma‐informed practices to use throughout the training and practicing these through role play.

New Veritas IPS staff and Orygen IPS staff (whose roles would be to supervise the new Veritas staff in IPS) participated in the one‐day TIC training program, developed as detailed above. The training was delivered by author W.P. in a group format by video link. Veritas IPS staff undertook the trauma‐informed training after completing standardised IPS training provided by an Orygen‐accredited IPS trainer. To support the implementation of TI‐IPS beyond the training day, Orygen IPS supervision staff were supervised monthly by W.P. in how to conduct TI‐IPS supervision. The supervision model was informed by a trauma‐informed supervision model for child protection workers (Oates 2022).

### 2.3 Qualitative Interviews

#### 2.3.1 Participants

Participants for the qualitative interview were recruited from young people who accessed the TI‐IPS program. To be eligible to participate in an interview, young people needed to have engaged in TI‐IPS for at least 3 months. Young people were excluded if they were unable to complete a half‐hour interview due to mental health, could not speak/understand English, or were diagnosed with a cognitive impairment. Ten young people were interviewed. Participant demographics are presented in Table 1.

**TABLE 1:** Participant demographics (*N* = 10).

Variable	*n*
Sex	
Male	4
Female	6
Age	
17 years	1
18 years	2
19 years	6
24 years	1
First Nations	
Yes	4
No	6
Country of birth	
Australia	10
Other	0
Relationship status	
In a relationship	6
Not in a relationship	4
Sexual orientation	
Straight	7
Bisexual	2
Undecided, not sure or questioning	1
Children	
Yes	2
No	8
Current accommodation	
House/flat with family of origin	4
Rented house/flat	4
Other (e.g., rented or free room)	2
Persons with whom living^a^	
Alone	5
Mother/step‐mother/foster mother	1
Father/step‐father/foster father	1
Partner	3
Grandparents/extended family	1

^a^ Participants were able to choose multiple options.

#### 2.3.2 Procedure

Ethics approval for the project was obtained from The University of Melbourne (Ethics no F22‐420). IPS workers verbally introduced the study and its aims and procedures to the young people they worked with, who had been actively involved in the IPS service for at least 3 months. They provided the young people who showed an interest in being interviewed with verbal and written information about the study. Young people who agreed to be interviewed were asked to sign the consent form. Signed consent forms were emailed to the interviewing researcher (I.K.), and a mutually convenient interview time was agreed. Participants received $30 for their time following completion of the interview.

#### 2.3.3 Data Collection

At the start of the interview, information about the study was repeated and young people were offered the opportunity to ask any questions. Verbal consent was confirmed and demographic information was collected. Participants were interviewed using semi‐structured interview guide developed based on the principles of trauma‐informed care was used (see Supplementary Table S1).

Because interviews about trauma have the potential to cause distress, I.K. was available for an additional 30 min if young people required debriefing. Young people who needed additional support could be referred back to their treating psychologist for immediate follow‐up. However, none of the young people experienced distress during their interviews and no further support was required.

#### 2.3.4 Research Team

The Orygen Trauma Team was responsible for all aspects of the research. W.P. developed the research protocol with input from young people (described above). W.P. provided training and supervision. I.K. conducted interviews, and data was analysed by I.K., S.B. and F.I. W.P. completed a PhD in trauma and youth mental health. She is an expert in organisational development and improvement initiatives. I.K. is an experienced researcher and psychologist. F.I. is a peer researcher in the trauma team at Orygen and has worked in volunteer roles in youth mental health services. S.B. is a senior researcher and experienced clinical psychologist working in the areas of childhood trauma, posttraumatic stress disorder and TIC in youth mental health. J.C. and J.K. are experts in service‐level implementation of IPS.

#### 2.3.5 Data Analysis

All interviews were audio recorded and transcribed. Data were analysed using reflexive thematic analysis (TA)—a method for identifying, analysing and reporting patterns within a qualitative dataset (Braun and Clarke 2006, 2021). We chose reflexive TA as it is a flexible yet robust approach that allows for data to be presented in a way that is accessible to a broad population (i.e., researchers, IPS workers, peer workers; Braun and Clarke 2006, 2014; Nowell et al. 2017). Our analysis was predominantly deductive, whereby data were analysed through the lens of key principles of TIC (e.g., trust, safety). However, we also endeavoured to ensure that participants' unique lived experiences of taking part in TI‐IPS were captured in our analysis. We aimed to achieve this through reflexive engagement with the data and viewing our subjectivity and diverse personal and professional backgrounds as a resource. This predominately involved (1) having two members of the research team with different backgrounds (I.K. and F.I.) review codes for a subset of transcripts, and (2) while analysing the themes as a group (I.K., S.B. and F.I.), exploring and discussing our different perspectives and individual reactions to clusters of codes and participant quotations.

We implemented the six phases outlined in Braun and Clarke's (2006) guidelines. Initially, I.K. familiarised himself with the data by reading the transcripts and taking notes about possible patterns within the data. He then conducted a manual process of coding by creating explanatory comments (codes). A two‐column table was created for each transcript whereby data was copied into the left column and codes written in the right column. To check whether the codes created by I.K. reflected the data (participants' words), F.I. evaluated three transcripts and their associated codes. All codes created by I.K. were maintained, and the wording of only a few codes was changed by F.I. I.K. then identified connections between codes and sorted them into themes. A coherent set of themes for the entire dataset was subsequently developed. To provide a rich interpretation of the data, throughout this phase I.K., S.B. and F.I. engaged in ongoing group discussions about the naming of themes and whether each theme reflected its associated codes. Finally, participants' quotations were selected to illustrate the developed themes. In the results section, participants are differentiated by an identification number ranging from 1 to 10.

Upon completion of this analysis, it was evident that some of our generated themes reflect well‐delivered standard IPS, which overlap with aspects of TIC (discussed in more detail in the Discussion section). While we acknowledge the importance of these findings, they are outside the aims of this study and have already been identified in the literature (Berring et al. 2024; Drake 1998). Therefore, in the results section we present a brief summary of these findings (the initial paragraph) and then provide a detailed outline of themes that describe (1) participants experiences of TI‐IPS that reflect key principles of TIC but are not central to IPS principles (e.g., safety), and (2) participants' experiences of talking about trauma with their IPS workers. Eleven themes are placed under three category headings that reflect key principles of TIC and relate to specific interview questions: (1) trust (and care), (2) safety, and (3) talking about trauma.

## 3 Results

All the young people reported that doing IPS at Veritas had generally been a positive experience. Young people described how IPS workers ‘individualise support’ (P10), and were ‘attentive’ and ‘considerate about other things going on’ (P1) in their lives. Young people mentioned that IPS sessions were often informal whereby they can ‘talk to them [workers] casually’ (P4). Young people described their workers ‘as very chill to talk to’ (P8) and ‘easy to get along with’ (P9). Most of the young people reported that their IPS workers would do more than what they were required to do and helped with things beyond education and employment. One young person mentioned that her IPS worker helped her ‘get a referral’ when her mental health was ‘completely shattered’ (P4), while another reported that her IPS worker has helped with ‘transport to get food and groceries’ (P10). All the young people indicated that they worked collaboratively with their IPS workers. Young people mentioned that they were ‘normally taking charge’ (P1) of the work, and that workers did not force them to accept presenting options (P9). Young people described how they felt able to freely share their opinions during IPS sessions. For example, they shared reflections such as, ‘she always asks me how I'm feeling about things’ (P2), ‘they always let me voice my opinion, and no matter what they always say my opinion matters’ (P4), and ‘I do tell her if I'm not comfortable with it or I can give it some thought, and she understands’ (P8).

### 3.1 Trust (and Care)

All the young people in the study said that they trusted their IPS workers.

#### 3.1.1 Trust Is Multifaceted

All the young people described different reasons for why they trusted their IPS workers. P1 and P7 mentioned that not feeling judged by their IPS workers was crucial to building trust, while P2 reported trusting his workers because they always look out for his best interests ‘no matter what’. P3 described trusting his IPS workers because they are reliable. He said ‘every time they say they can do something for me they've always been 100%… so it makes me feel like I can trust them’ (P3). P8 mentioned that she has built a trusting relationship with her workers because they treat her ‘more like an equal who just needs a bit of a push’ rather than someone who is ‘less than’. P7 emphasised that due to her past experiences she does not trust men and is thus ‘more comfortable’ with her workers and trusts them ‘because they're females’. Three young people (P2, P3, P7) mentioned that building trust was about knowing their IPS workers would keep their information private and confidential. For example, P2 said: ‘you can trust them to keep your problems to them and unless you really need help, then they won't tell anyone’.

#### 3.1.2 They Genuinely Care

Five young people (P1, P2, P3, P6, P10) described how they believe their IPS workers genuinely care about them. P2 reported that ‘there was a time where, like, I wasn't coping and she put off doing the work just to make sure I was ok’, and that ‘it just feels like she actually cares and supports me’. Young people mentioned that when their IPS workers showed genuine care about their progress towards achieving goals, they felt more motivated to help themselves. P10 indicated that her IPS worker shows that she is ‘interested’ by constantly talking to her and ‘asking for updates’. P10 said that this ‘motivates’ her to ‘try and get as much done as possible’. P3 said that ‘encouragement’ and talking ‘about how they [IPS workers] can help with it [getting his driving learner permit (L)]’ made him ‘feel like actually going for them’ [his L's].

### 3.2 Safety

All the young people in the study indicated that they felt safe when interacting with their IPS workers.

#### 3.2.1 It's When You Don't Feel Anxious or Threatened

Six young people (P1, P2, P7, P8, P9, P10) indicated that when they feel safe they don't feel anxious or threatened. P8 described feeling ‘mentally safe’ from ‘panic attacks’ during IPS sessions. P9 mentioned that feeling safe is when ‘you're not feeling like you need to watch out for anything’, and that she ‘definitely’ doesn't feel threatened ‘in spaces like that’ [during IPS sessions]. P7 described experiencing anxiety‐related physical changes when she feels unsafe. She said: ‘if I'm not in a safe situation I get like a real bad pit in my stomach. I'm fidgeting with like a ring’ (P7). P7 further described that while she feels safe with her IPS workers, when someone she doesn't know needs to attend a session (e.g., a new worker) her anxiety ‘kicks in’. This is largely because she is ‘overthinking everything’ (P7) and worrying about whether the person who she doesn't know is going to judge her. She emphasised the connection between being judged, safety and trauma: ‘the judgment part got to be a big play in it [feeling unsafe] because I've been like bullied my entire life’ (P7). P10 mentioned that ‘safe for me is like physical safety, safe from like harm from people… or putting myself in danger of, at risk of harm. I like to avoid that as much as possible’.

#### 3.2.2 They Care About Your Safety

Two young people (P2, P9) emphasised that their IPS workers care about their safety. P2 mentioned that because she has ‘a lot of trauma’ her worker will always make sure that she is feeling safe and comfortable, irrespective of whether their appointment is in the office or out in public. When talking on the phone, her worker will also ask about where she is and how she is feeling in her current location. P2 said: ‘It makes me feel so appreciated that she's willing to go the extra mile just to make sure I feel safe’.

#### 3.2.3 Letting Down Barriers

Two young people (P6, P10) reported that feeling safe was characterised by being able to open up when interacting with their IPS worker. P6 mentioned that he is ‘a very closed off person’, but when he feels comfortable and thus safe around someone he will ‘open up’. He said: ‘I'll just be myself. Like you can tell when I'm being fake and I'm not being fake [during IPS sessions]’ (P6). P10 spoke about feeling ‘safe enough’ to allow her worker to come to her home, and that she is ‘able to tell her stuff and she holds confidentiality quite heavily’.

### 3.3 Talking About Trauma

Eight young people reported speaking with their IPS workers about their trauma. However, six of these eight young people mentioned the trauma was not talked about often or in detail. Five young people mentioned that their IPS workers provided them with information about the effects of trauma.

#### 3.3.1 ‘I have therapists that help me with that bit’

The six young people who reported speaking minimally about trauma during their IPS sessions (P6, P2, P4, P6, P8, P10) also indicated that this is because they address trauma during counselling or other appointments with mental health services. P1 said: ‘Typically when I go there I don't really have that [trauma] on the agenda. If it was going to lean towards coming up, I do tend to avoid it. As I said, I keep them [trauma and IPS] separate’. Young people did mention that they felt comfortable talking about trauma with their worker, and if they were not engaging in therapy then trauma would likely be talked about more during their IPS sessions. P2 said that ‘it [trauma] would come up a lot more’ because she ‘wouldn't have another outlet’. P8 emphasised that she does currently have a ‘nice understanding of it [trauma]’ and that she does have her psychologist who is ‘there specifically for that support’. However, if she wasn't seeing a psychologist, she said: ‘it probably would come up more because I wouldn't have as great of an understanding of it as I do now’ (P8).

#### 3.3.2 It Gives Them a Better Understanding of Who You Are

Two young people (P6, P10) mentioned that talking about trauma was beneficial as it helped their IPS worker understand them better. P6 stated that when he commenced IPS he had an initial ‘big meeting’ where he explained ‘everything that's happened’ (his past trauma), and that it ‘was good to get them to get an understanding’. P10 reported that she believes there are ‘links’ between her trauma and current behaviour. Therefore, she likes ‘to explain’ why she does ‘something a certain way’ (P10).

#### 3.3.3 It's Beneficial Because There's no Judgement

Two young people (P2, P6) reported that disclosing trauma during their IPS sessions was a positive experience because they didn't feel judged by their worker. P2 stated that ‘it was a bit nerve wracking at the start’, but then ‘it was very helpful because there was no judgment from her [IPS worker] at all’ and it ‘felt nice to know that someone actually knew what was going on and I wasn't bottling it all up’. Regarding her worker's response, P2 further described: ‘it was how she never looked at me with like pity… and she never invalidated how I was feeling about everything’.

#### 3.3.4 Trust Makes It Easier to Open Up About Trauma

Two young people (P4, P5) indicated that talking about trauma was easier because they had built a trusting relationship with their IPS workers. P4 said: ‘At the time when I spoke about it, I knew I could trust them in listening and everything, so I basically ah, basically just had that chat with them’. Building initial trust was thus beneficial for P4 because she was ‘able to talk to them’ and ‘release whatever feelings’ she was having. Similarly, P5 reported that while disclosing trauma was ‘kind of hard’, it ‘felt good’ to talk with someone he could trust. He further described how his worker made sure that he wasn't overly distressed and didn't force him to disclose his entire story: ‘He offered me like a break and stuff, and then he told me I don't have to continue if I don't feel comfortable with it. And he didn't force me to say it all’ (P5).

#### 3.3.5 Talking About Trauma Is Distressing and Thus Avoided

One young person (P7) reported that she avoids talking about trauma with her workers and gets ‘overwhelmed’ and ‘really uncomfortable’ when asked questions about past traumatic events. She did mention that her current IPS workers can tell by her body language if she is distressed and she can inform them if she is feeling uncomfortable. She emphasised that her workers ‘haven't really gone in depth’ about the impact of trauma on mental health because she does not want to listen. When her workers have spoken to her about trauma she feels it may lead to them suggesting she try counselling (which she doesn't want to do) and thus feels they are ‘judging’ and ‘not respecting’ her in those moments (P7).

#### 3.3.6 Gaining Insight

Four young people (P2, P4, P5, P6) reported that having conversations with their IPS worker and/or being provided with written information about the effects of trauma was incredibly beneficial. P2 mentioned that while she predominantly talked about trauma in therapy, engaging in discussion with her IPS worker about how sexual abuse could affect her if she was studying or working was ‘very helpful’ as it offered ‘a different perspective on everything’. She said:

There was a time when we were speaking about sexual abuse, and she was saying how that can affect my day‐to‐day life, I can't remember the exact words she said but the way she would word it would make it make so much sense to me (P2).

P5 described how he always thought he was ‘different to the other people’ and that in the past he was ‘a really angry person’. He mentioned that his IPS worker spoke to him about how a person's past can be reflected in their present and future, which helped him understand how his anger was related to past trauma. P4 reported that having conversations with her workers about the effects of trauma was ‘really helpful’ because ‘it was easier to know how to get the help… or even just speak to them’. P6 stated that it was helpful to receive brochures which acknowledged the impact of trauma on young people:

Not many people like to acknowledge that a lot of young people have gone through some stuff and they just kind of think we're just acting out. But when I got those brochures it was like kind of like clear that I'm not this way because I want to be this way (P6).

## 4 Discussion

This study is the first to our knowledge to explore trauma‐exposed young people's experience of participating in an IPS program where workers had their training and supervision augmented with a model of trauma‐informed care. We found that, when asked in‐depth questions about their experience of the trauma‐informed IPS program, young people described how important trust in the IPS worker was and how trust supported them in being motivated to engage in looking for work. They also described feeling safe interacting with their IPS worker, a key feature of trauma‐informed care. Trust in, and safety with professionals have been found to be central for trauma‐exposed young people when engaging in mental health support (Ellinghaus et al. 2021; Tong et al. 2019; Truss et al. 2022). Further, we found several instances where young people talked about trauma with their IPS workers in a brief and contained way, and young people experienced this as helpful and supportive.

### 4.1 Similarities Between IPS and TIC

In analysing the results of this study, we discovered that many of the ingredients of TI‐IPS identified as important by young people exposed to trauma were common to both IPS and TIC. These were being offered personalised, flexible, holistic, informal, collaborative, and engaged support (Berring et al. 2024; Drake 1998). These were not described in detail in the results section as they are already central to both TIC and IPS, and therefore relatively unremarkable in that regard. However, it appeared that the two models were able to be integrated without losing the central principles of each.

During TI training and supervision, IPS workers were furnished with a clear position on their role as regards talking about trauma with the young people they were working with. This was important because we had heard in previous consultations on how services should address trauma in the youth mental health sector that non‐clinical staff had a lack of clarity on their role as regards trauma (Bendall, Phelps, et al. 2018). While the remit of non‐clinical staff often does not include talking about trauma, when these staff use a youth‐centred model with low formality and a focus on relationship, the young people they work with often bring up trauma. However, this means non‐clinical staff are not equipped or empowered to manage these situations, potentially leading to role confusion and vicarious trauma. One of the key principles of trauma‐informed care is a service must be able to *Respond* to trauma (SAMHSA 2014) so furnishing non‐clinical staff with appropriate ways to do this essential. Several aspects of the TI‐IPS training model were developed to address this. Firstly, IPS workers were given guidance on what to do when young people brought up trauma. This was based on the model for psychological first aid that was developed for the general population to support them to respond to people impacted by trauma (Wang et al. 2021). Secondly, they were given information about the effects of trauma on young people in combination with online youth‐friendly resources with the same information that they could give directly to young people. This included how trauma could affect young people in the workplace. This enabled them to respond to trauma in two ways: by giving information and using principles of psychological first aid. The results of the study show that, at least for the young people in the study, the training and supervision were successful in balancing the understanding that, while IPS was not the place for focus on trauma, IPS workers were comfortable and helpful in talking about trauma if it came up. Importantly young people said that building a trusting relationship, key to IPS, encouraged them to talk about trauma. This is consistent with young people's experiences of working with clinical staff where they state that ‘opening up’ about trauma can only happen in an environment of trust and care (Dryden‐Mead et al. 2022; Tong et al. 2019). As we transform our mental health services to include more non‐clinical staff (such as vocational workers and peer workers), and they build connection and trust, young people will want to talk about trauma with them. We must make sure we have supports in place for this to happen in a trauma‐informed way to support both young people and staff.

One of the key aspects of TIC in youth services is that there is interagency collaboration or team‐based care within organisations (Bendall et al. 2021). This enables all those helping a young person to have a shared understanding of the challenges they face, their strengths, and how these connect with their past and current context. There was evidence that the presence of good quality multi‐disciplinary care for the young people in this study supported IPS workers in being able to use the TI‐IPS model to address trauma to an appropriate degree. Some young people suggested that if they did not have a therapist to talk to about their trauma, they would have asked for more intense support around trauma from their IPS worker. This highlights the importance of multi‐disciplinary care for young people exposed to trauma (Bendall, Phelps, et al. 2018). Importantly, this team‐based approach to supporting young people with their social, vocational, and mental health needs may not be possible in all settings. In many systems, IPS is external to mental health services and it is unclear how integration of a trauma‐informed approach could occur in these systems (Bonfils 2022; Moe et al. 2021). Surveys of staff and young people in these settings could lead to better understanding.

Services for adults with serious mental illness are moving towards more trauma‐informed models of care, which gives service users greater permission to disclose and appropriately explore their trauma experiences with their workers. As this occurs, it is important for IPS workers in adult mental health settings to receive trauma‐informed care training to help them to have containing conversations about trauma. This will support the safety of both service users and IPS workers. More research is needed on the optimal model for this across both young and adult mental health service delivery.

The aims of this project were to develop and implement TI‐IPS in a service in Australia and, importantly, to test an early version of our training and supervision model to understand how young people experienced receiving TI‐IPS. A key reason for this early testing was to have young people's direct experience of this service innovation shape the early development of the training model. We consider this to be part of keeping young people at the centre of the development of services, alongside consultation with youth advisors and employment of youth peer researchers.

### 4.2 Strengths and Limitations

This program was implemented in the context of a trauma‐informed service, which was able to offer multi‐disciplinary care to young people. It is unclear whether young people would have had the same experience with TI‐IPS if it had not occurred within the context of this wrap around care. Further, we did not interview TI‐IPS workers about their experiences of delivering TI‐IPS and other Veritas staff on their views on the role of the TI‐IPS workers. These would have strengthened our knowledge of the feasibility and acceptability of TI‐IPS. Young people were informally invited to participate in the study by their IPS worker. A strength of this informal recruitment method was that we were able to leverage the trust young people had in their worker to gain their trust to engage in a research interview. Young people in transient accommodation, and with complex psycho‐social needs and priorities, are traditionally very hard to engage in research. A weakness of the method was that it did not allow for systematic collection of data on the number of young people approached, the proportion who agreed, and the reasons for decline. It also did not capture young people who disengaged or may have been dissatisfied with the service. Another strength of the study was the inclusion of youth and lived experience expertise in the project team. This could have been strengthened further by the presence of a lived experience researcher in the conceptualisation of the study.

## 5 Conclusion

The trauma‐informed and IPS models of care were able to be integrated successfully into the Veritas House service model and young people highly valued the relationship with their worker when undertaking TI‐IPS. These findings highlight the importance of a trauma‐informed lens on all aspects of youth mental health service delivery.

## Funding

This work was supported by the Australian Government.

## Conflicts of Interest

The authors declare no conflicts of interest.

## Supporting information


**Table S1:** Interview guide.

## Data Availability

Research data are not shared.
