# Supplementary material for: Trauma‐Informed Individual Placement and Support in a Youth Community Service in Regional Australia: Trauma‐Exposed Young People's Experiences
Source: Early Interv Psychiatry. 2026 Oct 16;20(10):e70271. doi: 10.1111/eip.70271 (PMC13673282; doi:10.1111/eip.70271)
Supplement: Supplementary file 1 [file EIP-20-0-s001.docx]

**Supplementary Table 1: Interview Guide**

1. Tell me about your experiences of doing IPS at Veritas?
   i. What did you find most /least useful?
2. What was it like working with [insert IPS worker name] [prompt principles; trustworthiness and transparency, collaboration; empowerment, choice]
   i. *Possible prompts:*

*-*What was good about it?

*-*What was bad about it?

*-*What did you like her doing?

*-*What did you not like her doing?

ii. *Follow up question*: She made you feel [descriptive; e.g., comfortable, motivated, worked collaboratively], what does that mean?

-Do you feel comfortable working with xxx?

-Are you able to give your opinion about what you do together, do you work collaboratively?

We are doing this research study because we are trying to work out how best to help young people who have experienced trauma or other major difficulties to get into education or employment.

3. We are really interested in how safe you felt when working with xxx. Did you feel safe working with xxx?

Possible prompts:

-What does it mean to be safe?

-Did you ever talk about trauma or other major difficulties, what was that like?

-Did you ever feel (di)stressed in an IPS session, what happened?

-Were there times doing IPS that were more difficult than other times? *Tell me more about that time?*

4. *Did xx talk with you about the effects of trauma or the difficulties that you have been experiencing? What was that like?*
5. Would you recommend the IPS program to your friend who had experienced trauma? if so why? Why not?
6. Is there anything else you would like to discuss that we have not spoken about?
